# New remote centre of motion mechanism for robot-assisted minimally invasive surgery

**DOI:** 10.1186/s12938-018-0601-6

**Published:** 2018-11-20

**Authors:** Xiaoqin Zhou, Haijun Zhang, Mei Feng, Ji Zhao, Yili Fu

**Affiliations:** 1 Jilin University, Nan Guan District, Changchun, China; 2 Harbin Institute of Technology, Nan Gang District, Harbin, China

**Keywords:** Robot-assisted minimally invasive surgery, Remote centre of motion mechanism, Motion error

## Abstract

**Background:**

Robot-assisted minimally invasive surgery (RMIS) is promising for improving surgical accuracy and dexterity. As the end effector of the robotic arm, the remote centre of motion mechanism is one of the requisite terms for guaranteeing patient safety. The existing remote centre of motion mechanisms are complex and large in volume, as well as high assembly requirement and unsatisfactory precise. This paper aimed to present a new remote centre of motion mechanism for solving these problems.

**Methods:**

A new mechanism based on the RMIS requirements is proposed for holding the laparoscope and generating a remote centre of motion for the laparoscope. The mechanism kinematics is then analysed from the perspective of the structural function, and its inverse kinematics is determined with a small number of calculations. Finally, the position deviation of the laparoscope rotational point is chosen as the index to evaluate the mechanism performance. The experiments are performed to test the deviation.

**Results:**

The position deviations of the laparoscope rotational point do not exceed 2 mm, which is lower than that of the existing remote centre of motion mechanism. The 2 mm positioning error of the laparoscope won’t affect surgeon observation of the surgical field, and the pressure caused by the positioning error was acceptable for the skin elasticity. The proposed mechanism meets the RMIS requirement.

**Conclusions:**

The proposed mechanism can achieve the remote centre of motion for the laparoscope. Its simple and compact structure is beneficial to avoid the collision of robotic arms, and it can be applied on other robots for providing the instrument necessary motion in minimally invasive surgery.

## Introduction

Robot-assisted minimally invasive surgery (RMIS) has had a revolutionary impact on surgery, having the ability to satisfy the requirements of higher precision and dexterity for surgery operations. In traditional minimally invasive surgery (MIS), a surgeon holds instruments to perform a surgical operation [1]. In RMIS, the instrument is operated by a robot manipulator to penetrate the patient’s body and perform surgical operations (e.g., cutting, tying, and suturing) [2, 3]. Under the constraint imposed by the ‘minimally invasive’ incision, two tangential motions of the instrument must be confined at the incision port to ensure patient safety [4]. Hence, the instrument has four degrees of freedom (DOFs), namely, pitch, translation, roll, and yaw [5]. For convenience, the concept of the remote centre of motion (RCM) has been devised to describe the pitching and yawing movements around the incision port [6], and RCM generation is one of the requisite terms for an MIS robot that is directly related to patient safety.

Many researchers and institutes have conducted studies on the generation of RCM-based motion. There are two representative techniques: a control method for creating a virtual RCM [7–14], and a mechanical method for creating a physically constrained RCM [15].

The control method employs software algorithms to generate a virtual RCM. This approach can be applied regardless of the robot structure and has the great advantage of robot design simplification. However, it is difficult to guarantee safety in the case of electronic-component or power malfunction; thus, this method is rarely used in commercialised models [5].

The mechanical method employs special mechanisms to provide RCM-based motion for surgical instruments. This approach is more reliable than the previous method, as the injury risk from unexpected control failures is inherently minimised by the structure [16]. The mechanisms to perform RCM-based motion are collectively called ‘RCM mechanisms’ [17, 18] and utilise parallelograms, spherical linkages, gear trains, etc. The characteristics of each RCM mechanism are described in the following.

The parallelogram mechanism is commonly adopted in MIS robots having high rigidity and, such as the Neurobot [19], BlueDRAGON [20], and the famous da Vinci Surgical System (Intuitive Surgical Inc.) [21]. In addition, many manipulators have been developed based on this architecture, which have diverse structural forms [5, 20, 22–32]. However, a conflict exists between the mechanism movement range and structure. Because the distance between the two transverse bars of the parallelogram becomes shorter when the mechanism attains an extreme angle, the two transverse bars should be mounted far from each other to prevent overlapping. This requirement yields a large-volume structure that does not effectively prevent multi-robotic arm collision. In addition, the RCM position is affected by the relative positions of the upright and transverse bars.

Using the geometric features of circles and spheres, researchers have developed both circular arc [33–38] and spherical [39–46] mechanisms. The installed instrument’s axis passes through the mechanism arc or sphere centre, which is set to coincide with the incision port during surgery, thus the instrument can be steered to rotate around the incision port. These types of mechanisms have a small structure; however, this structure is specialised and, thus, high processing and assembly precision are required. In addition, these mechanisms are mainly used to hold lightweight instruments, because of their slightly low rigidity [46, 47].

Lehman et al. [48] have assembled several bevel gears in combination, with the gear axes passing through the incision port. Thus, the driving gear rotation causes the instrument to rotate around the incision port. However, gear clearance exists for gear drive, which has an influence on the control precise of the instrument movement. In addition, the gears require lubrication, which has a negative effect on sterilisation [49].

Li et al. [50, 51] have used three identical CRRR structures to construct a mechanism, where C denotes a cylindrical joint and R a revolute joint. The mechanism employs linear actuators and benefits high rigidity and load capacity from the parallel structure. This parallel mechanism has provided new perspectives for achievement of RCM-based motion. However, the parallel mechanism has a large volume, rendering this design unsuited to surgical operation requiring multi-robotic arm cooperation.

To sum up, the software method has lower security than the mechanical method to achieve RCM-based motion in the case of electronic-component or power malfunction, however, the existing RCM mechanisms are complex and large in volume, as well as high assembly and machining requirement. In view of the importance of safety for MIS, this paper reports realisation of RCM-based motion using the mechanical approach. Considering the contradiction between stiffness and volume in current RCM mechanisms, a planar symmetrical-rod structure in the parallel form is proposed to generate RCM-based motion. Combined with a simple axis-driving joint in series, a new 2-DOF RCM mechanism is presented that merges the advantages of parallel architecture, decoupled motion, and a simple design. Its simple structure is convenient as regards machining and assembly. Moreover, the RCM location is independent of the joint relative position; therefore, repeat adjustment of the joint initial posture for calibration of the RCM position is unnecessary. Because of its compact and small structure, the proposed mechanism can be applied to multi-robotic arms as the end effector to provide the necessary instrument motion and can effectively prevent collision between the robotic arms.

## Materials and methods

### Mechanical structure design

In a traditional minimally invasive surgery, two assistants always help the surgeon accomplish a surgical operation: one holds the laparoscope to provide the surgeon with a visual display of the surgical site, while the other holds surgical instruments to perform some auxiliary work. For example, when the surgeon needs to cut a particular part of the body, the assistant uses an instrument to elevate the part to facilitate the cutting operation, because the tissues and the organs have a soft texture. The assistant may become tired, especially in some major surgeries, where instruments must be held for long durations. Consequently, the movements performed by hand may not provide sufficient stability, thereby affecting the surgical visual display or the tissue boundary dissociation.

**Fig. 1:**
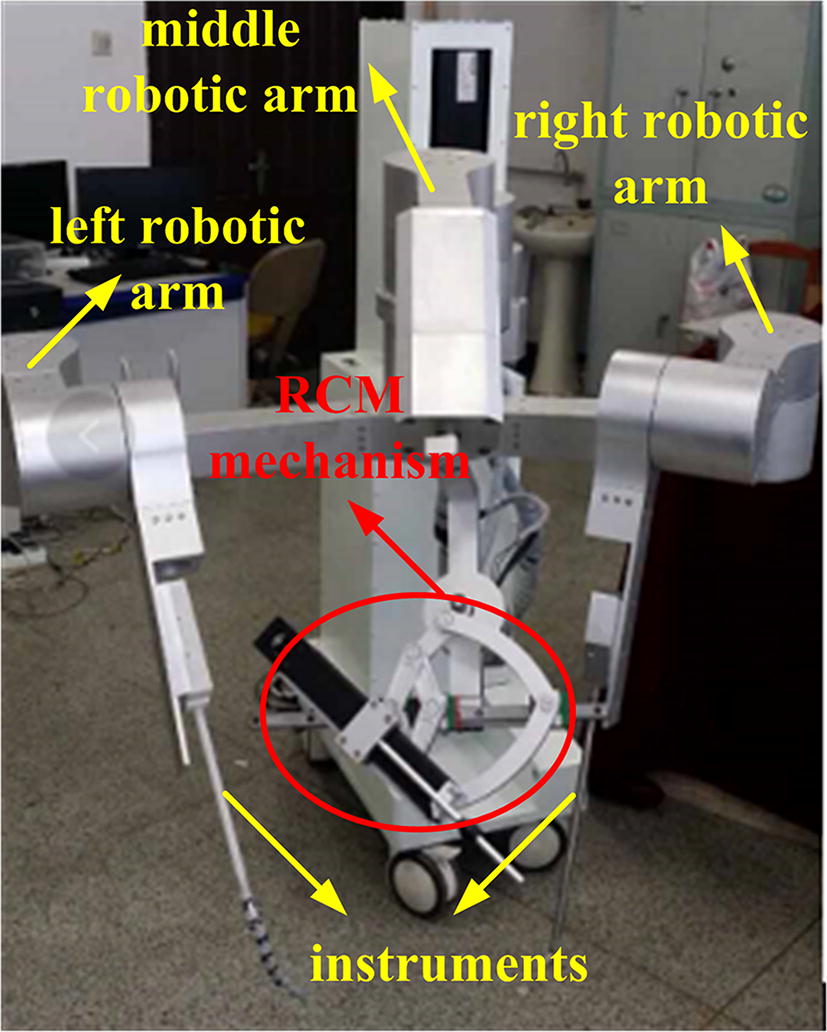
Custom-designed robot

**Fig. 2:**
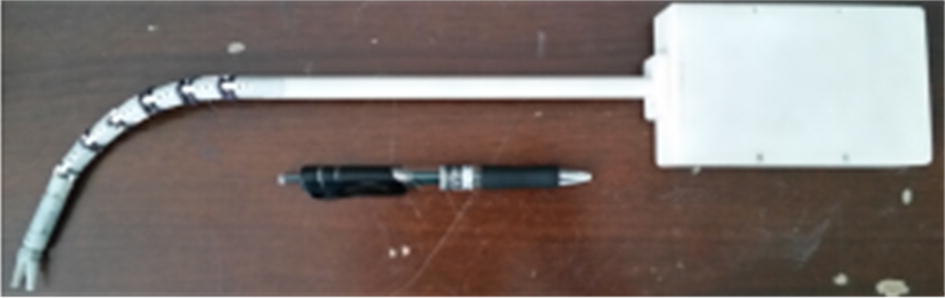
Flexible surgical instrument

We custom designed a robot that could perform the assistant’s work to overcome this problem. As shown in Fig. 1, the robot had three arms: the middle arm and the left and right arms. The middle arm was used to hold the laparoscope and provide a visual display of the surgical site. This arm consisted of one linear joint, three positioning joints, and an RCM mechanism. The linear joint adjusted the height of the robotic arm relative to the operating bed, while the positioning joints enabled the laparoscope to reach the incision port. The RCM mechanism, which served as the end effector of the middle arm, held the laparoscope and facilitated its position change without causing pain at the incision port. The left and right arms were used to hold surgical instruments to accomplish auxiliary work. Unlike the laparoscope, the surgical instrument was designed to be flexible. As shown in Fig. 2, the instrument had a snake-like configuration with wire actuation, which allowed the instrument to achieve a position change in the patient’s body. Thus, the left and right arms did not need to have a RCM mechanism. Therefore, the key points of the robot development lay in the flexible design of the surgical instrument and the RCM mechanism. This study focused on the RCM mechanism design.

**Fig. 3:**
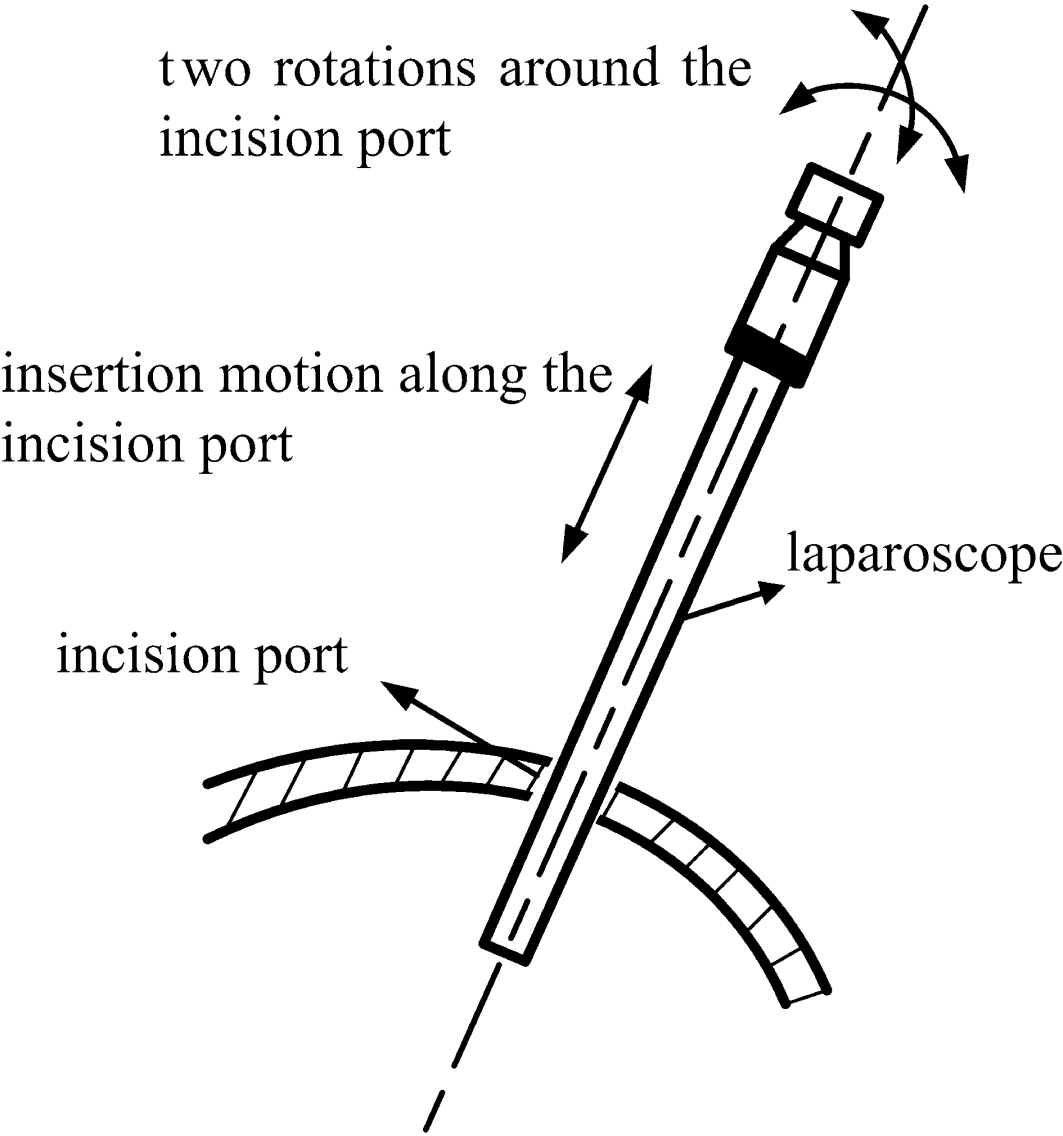
Illustrative diagram of laparoscope motion

The RCM mechanism was used herein to hold the laparoscope and provide its motion for the surgical visual display. As mentioned earlier, the movements along the incision tangential direction were constrained to ensure patient safety. The visual display does not need to be rotated during surgery; thus, the motions of the laparoscope were left as shown in Fig. 3 (i.e., two rotations (pitching and yawing) around the incision port and one insertion motion along the incision port). Therefore, the RCM mechanism was designed to achieve the pitching and yawing motions and the insertion movement of the laparoscope.

**Fig. 4:**
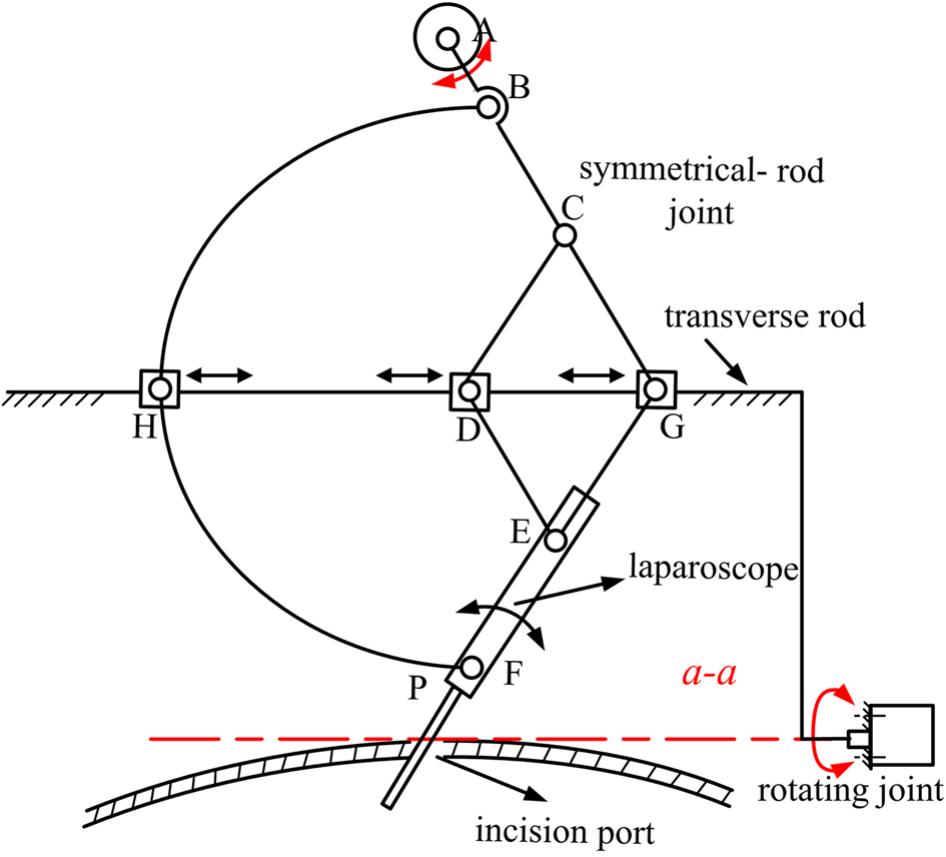
Illustrative diagram of RCM mechanism

A new RCM mechanism was proposed according to the abovementioned requirements. The mechanism consisted of a rotating joint, a symmetrical-rod joint, and a linear joint (Fig. 4). The three joints were connected in series: the symmetrical-rod joint was connected to the end of the rotating joint; the linear joint was connected to the symmetrical-rod joint; and the laparoscope was mounted on the linear joint. The rotating joint was an axis-driving joint (Fig. 4). Its *a*–*a* axis passed through the incision port; thus, its rotation moved the laparoscope in a yawing motion around the incision port with no pressure.

The symmetrical-rod joint had a symmetrical structure (Fig. 4) and consisted of rods AC, CG, CD, DE, EG, EF, BH, and HF. The rods were hinged together and were symmetrical to the transverse rod, along which hinge points D, G, and H could slide. Rod AC was connected to the rotating joint by hinge point A and actuated by a motor. Hinge point A was located on the *a*–*a* axis and could be treated as a fixed point relative to moving rods. The velocity values of hinge points E and F were equal to those of hinge points C and B, respectively, because of the symmetrical structure. Therefore, the laparoscope maintained the same motion with rod AC. Point P, which was the intersection of the *a*–*a* axis and the laparoscope, was symmetrical to and had the same velocity as hinge point A. Thus, the velocity of point P was zero. It could also be seen as a fixed point. During surgery, point P was set to coincide with the incision port; hence, the rotation (pitching) of the laparoscope was around the incision port, and no pressure could be applied on the incision port. To summarise, the proposed mechanism can output a fixed rotational centre with no pressure on the incision site. Moreover, the location of point P depends on the hinge point A’s position according to the restriction provided by the structure’s symmetrical relationship, which is not affected by the initially assembled mechanism posture. Note that linear joint responsible for the laparoscope-insertion movement was a ball–screw pair, which is omitted from Fig. 4 as it has the ‘as-known’ structure.

**Fig. 5:**
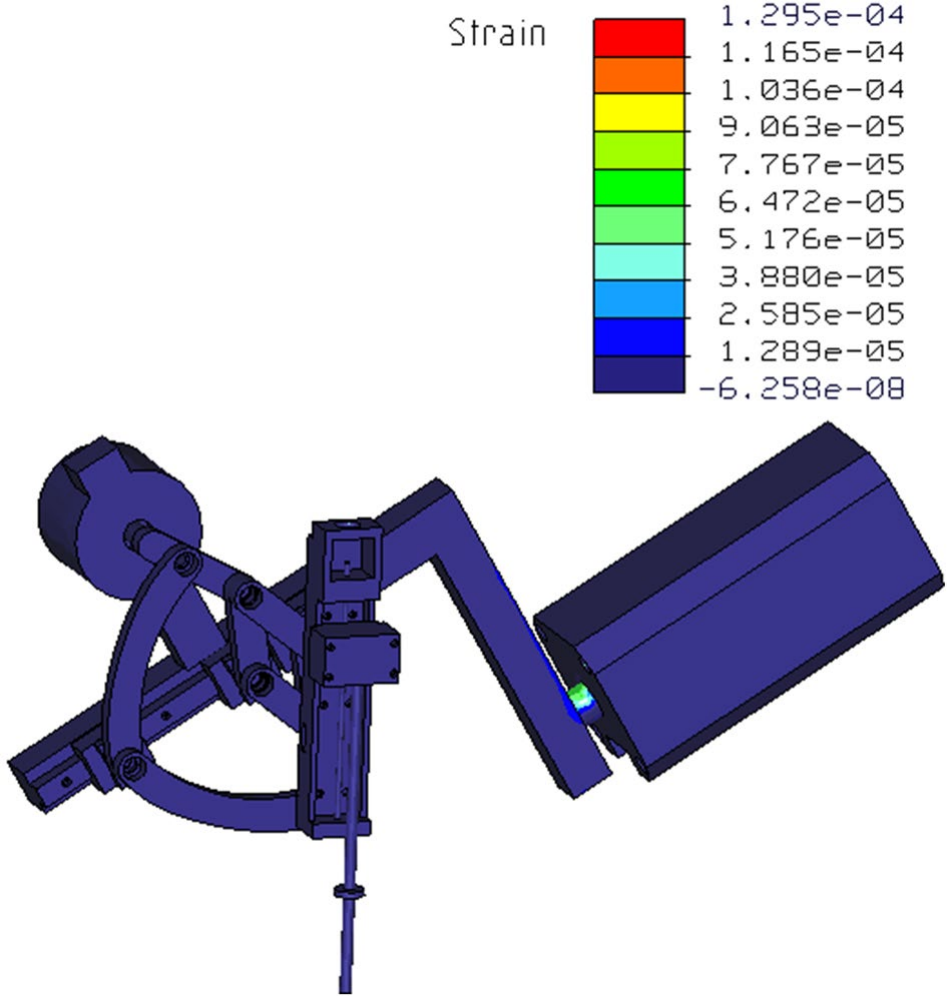
Result of finite element analysis of RCM mechanism

In this paper, the RCM mechanism was used to hold laparoscope for providing the surgery visual display, different from surgical instrument, laparoscope had no force interaction with surgical site, so the loads for this RCM mechanism were the laparoscope gravity and the friction force from trocar port. In this condition, the structural strength was analyzed with finite element method as shown in Fig. 5, which demonstrated the RCM mechanism had good static stability.

According to the RMIS characteristics, the proposed RCM mechanism has two working modes: passive and active. The motion of the mechanism in the passive mode is driven by the dragging motion of the surgeon to adjust the laparoscope posture, which is helpful in saving the adjustment time required for configuring the preoperative settings. Meanwhile, in the active mode, the movement of the RCM mechanism is actuated by motors, and the rotation angles of the motors are acquired through calculation of the inverse kinematics using the movement information pertaining to the end of the laparoscope. The kinematics of the RCM mechanism was discussed in the following section.

### Kinematic analysis of RCM mechanism

**Fig. 6:**
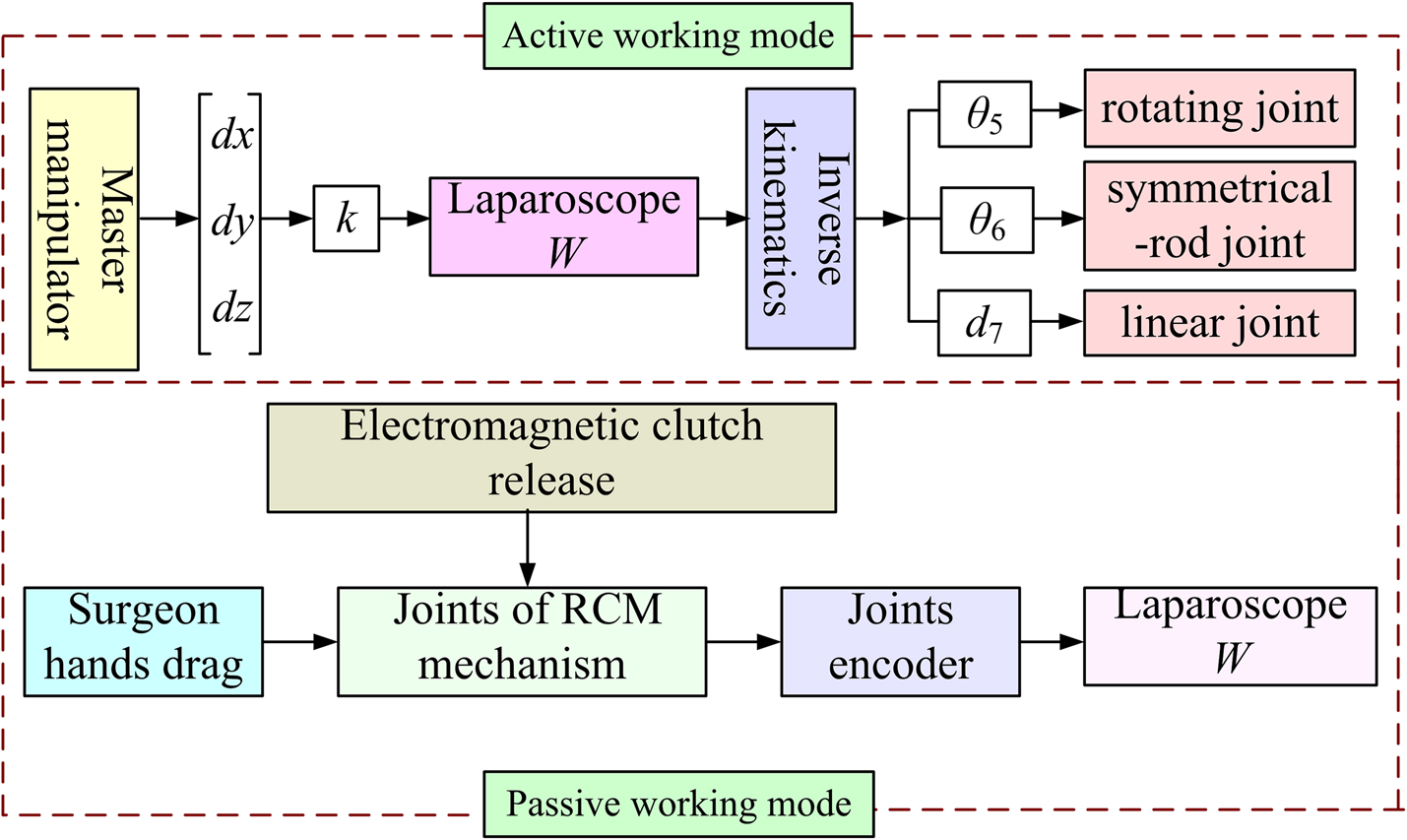
Control block diagram of the RCM mechanism

In RMIS, the surgeon uses a pair of master manipulators to control the robot movement. The robot makes the laparoscope (or the instruments) move synchronistically with the master manipulators. In this manner, the operation of the surgeon’s hands is reproduced by the robot. Therefore, the information pertaining to the operation of the surgeon’s hands should be extracted to control the robot movement by collecting the movement information of the master manipulators. In this study, position control of the laparoscope only is needed, because the proposed RCM mechanism is used to hold the laparoscope. In the active mode, the movement of the master manipulators described by *dx*, *dy*, and *dz* in Fig. 6 is mapped to the laparoscope as the laparoscope position *W* in every sampling cycle, considering the fact that the movement of the laparoscope is provided by the RCM mechanism, to reproduce the operation of the surgeon’s hands. Thus, the inverse kinematics should be solved to control the laparoscope movement and determine the range of the joint movement of the RCM mechanism. Figure 6 shows the control strategy, where *k* is the scaling factor used to scale the movement of the surgeon’s hands. For pre-operative setting, to save the adjustment time, the mechanism worked in passive mode, the electromagnetic clutch released, the surgeon dragged the RCM mechanism to make the laparoscope locate above the surgical site, in this process the joints encoders recorded the joints rotation angles, which were used for the calculation of the laparoscope position *W* by using the following forward kinematics.

**Fig. 7:**
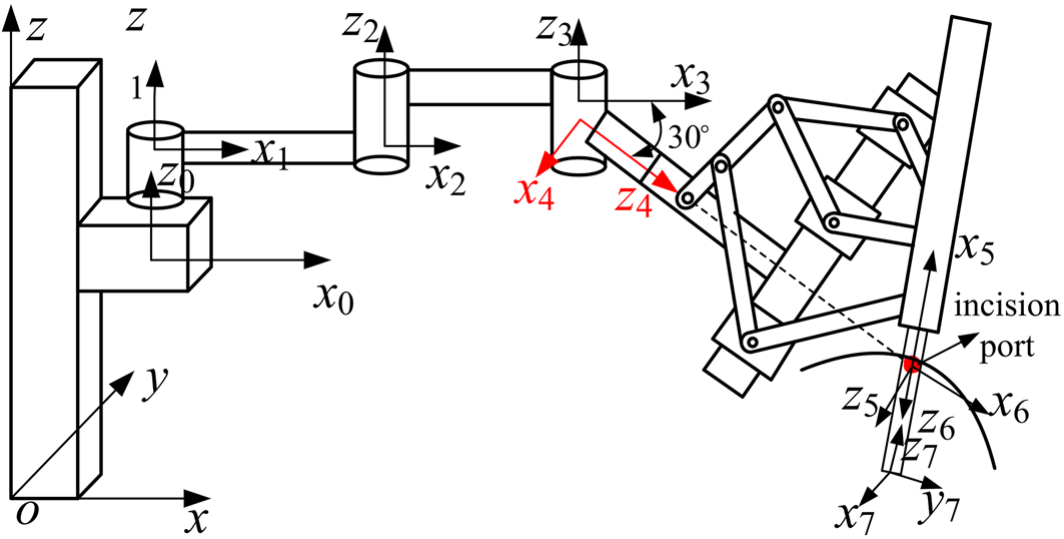
Coordinate systems of robotic arm

The laparoscope was held by the RCM mechanism; hence, coordinate systems of the middle arm joints were built using the Denavit–Hartenberg (D–H) method to describe the laparoscope position in the robot coordinate system. The function of the RCM mechanism was to provide the pitching and yawing movements of the laparoscope around the incision port as well as the insertion movement along the incision port. Thus, to simplify the establishment of the forward kinematics model, we built the coordinate systems of the RCM mechanism joints from the perspective of a structural function, such that the joints could be treated as two rational joints and one linear joint located at the incision port (Fig. 7). Table 1 presents the kinematic parameters of the middle arm joints.

**Table 1:** D–H parameters of RCM mechanism

Joint	*a* (mm)	*α* (°)	*d* (mm)	*θ* (°)
1	0	0	100	0
2	270	0	0	*θ*_2_
3	150	0	0	*θ*_3_
4	0	− (90 + 30)	0	*θ*_4_
5	0	− 90	620	*θ*_5_
6	0	90	0	*θ*_6_
7	0	0	*d*_7_	0

As mentioned earlier, once the laparoscope reaches the incision port, the positioning joints of the robotic arm are locked during surgery. The homogeneous coordinate transformation matrix ^*o*^T_*o*4_ of the coordinate system *o*_4 _− *x*_4_*y*_4_*z*_4_ of the rotating joint relative to the base coordinate system *o*− *xyz* is a constant, which can be obtained using the feedback from the positioning joint encoders. Thus, to facilitate the calculation, the laparoscope position *W* in the coordinate system *o*–*xyz* can be converted to the position *W’* in the coordinate system *o*_4 _− *x*_4_*y*_4_*z*_4_, as follows:

1 $$ W^{\prime}{ = (}{}^{o}{\text{T}}_{o4} )^{ - 1} W $$

Considering that the changes in the laparoscope position are provided by the movements of the RCM mechanism joints, the relationship between the joint movements and the laparoscope position can be described as follows:

2 $$ {}^{5}T_{4} \cdot {}^{6}T_{5} \cdot {}^{7}T_{6} = W^{\prime} $$

where ^j^*T*_i_ is the homogeneous coordinate transformation matrix of the coordinate system *i* of joint *i* relative to the coordinate system *j* of joint *j*.

The inverse kinematics solution of the RCM mechanism joints 5–7 is obtained as follows to solve Eq. (2):

3 $$ \left\{ {\begin{array}{*{20}l} {\theta_{5} = \arctan \left( {\frac{{W_{y}^{\prime } }}{{W_{x}^{\prime } }}} \right)} \\ {\theta_{6} = \arctan \left( {\frac{{W_{y}^{\prime } }}{{\sin (\theta_{5} ) \cdot (W_{z}^{\prime } - 620)}}} \right)} \\ {d_{7} = \frac{{W_{z}^{\prime } - 620}}{{\cos (\theta_{6} )}}} \\ \end{array} } \right. $$

where *θ*_5_, *θ*_6_, and *d*_7_ are the ranges of motion of the rotating, symmetrical-rod, and linear joints, respectively. There are no coupling motion of the three joints.

### Experiments

As mentioned earlier, the RCM mechanism is used to hold the laparoscope and provide its pitching and yawing movements around a point. This point is set to coincide with the incision port to alleviate stress and ensure patient safety, and should be a fixed point to keep the coincidence with the incision port when the laparoscope moves. Thus, the “fixed” point is the testing point and its the position deviation is used herein to evaluate the performance of the proposed mechanism and verify the effectiveness of the proposed mechanism for achieving RCM-based motion. For convenience, its initial position is referred to as *P*_0_ (*x*_0_, *y*_0_, *z*_0_), and the collected position during the mechanism movement is referred to as *P*_*i*_ (*x*_*i*_, *y*_*i*_, *z*_*i*_). Therefore, the deviation between *P*_0_ and *P*_*i*_ is as follows:

4 $$ e_{i} = \left| {P_{i} - P_{0} } \right| = \sqrt {(x_{0} - x_{i} )^{2} + \left( {y_{0} - y_{i} } \right)^{2} + \left( {z_{0} - z_{i} } \right)^{2} } \quad i = 1,{ 2},{ 3,} \ldots {\text{n}} $$

where the deviation *e*_*i*_ represents the position change between the collected position *P*_*i*_ and the initial position *P*_0_, and subscript *i* represents the serial number of the collected position.

**Fig. 8:**
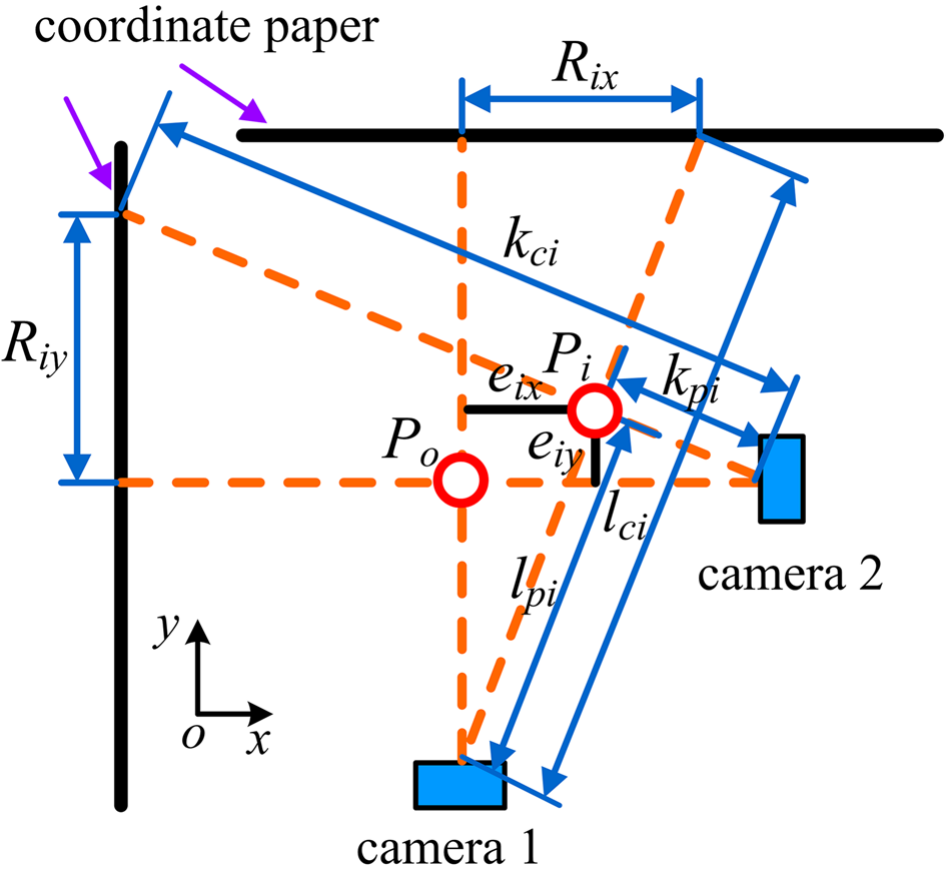
Illustration to test position deviation in experiment

We replaced the laparoscope with a tube having the same diameter to test the position change of the point around which the laparoscope rotates. The movement of the tube was divided into steps, and we used two cameras to test the position change *e*_*i*_ in every step. Figure 8 illustrates the measuring principle. First, the rotating and symmetrical-rod joints were set to their initial positions to mark *P*_*o*_ (Fig. 8). Two coordinate sheets were then placed vertically, and two cameras were set at the location, where the lines between the camera lenses and *P*_*o*_ were vertical to the coordinate sheets. Thus, the *e*_*i*_ of the collected position *P*_*i*_ in every step could be acquired by its projections (*R*_*ix*_, *R*_*iy*_) on the coordinate sheets. The distances between the cameras and *P*_*i*_ and those between the cameras and the projections on the coordinate sheets in every step were measured. The components of *e*_*i*_ can be calculated as follows:

5 $$ \left\{ \begin{aligned} & e_{ix} \, = \,R_{ix} \cdot \frac{{l_{pi} }}{{l_{ci} }} \\ & e_{iy} \, = \,R_{iy} \cdot \frac{{k_{pi} }}{{k_{ci} }} \\ \end{aligned} \right. $$

where *e*_*ix*_ and *e*_*iy*_ are the components of *e*_*i*_ along the *x*- and *y*-axes, respectively; *R*_*ix*_ and *R*_*iy*_ are the projections of *e*_*i*_ on the coordinate sheets; *l*_*pi*_ and *l*_*ci*_ are the distance between camera 1 and *P*_*i*_ and that between camera 1 and the projection on the coordinate sheet, respectively; and *k*_*pi*_ and *k*_*ci*_ are the distance between camera 2 and *P*_*i*_ and that between camera 2 and the projection on the coordinate sheet, respectively.

Thus, the *e*_*i*_ of the *P*_*i*_ in every step can be obtained as follows:

6 $$ e_{i} \, = \,\sqrt {e_{ix}^{2} \, + \,} e_{iy}^{2} $$

**Fig. 9:**
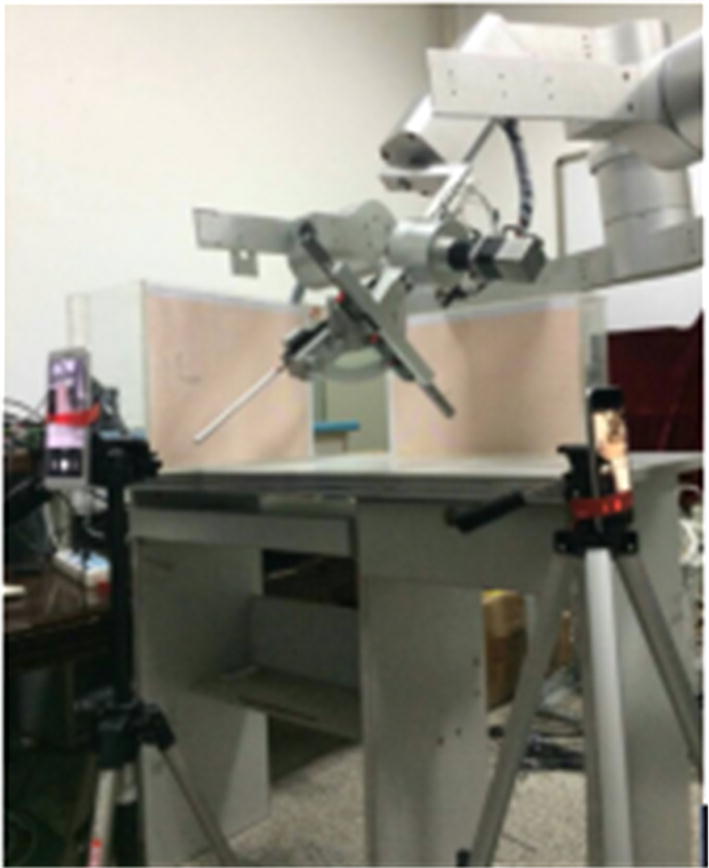
Experimental setup for evaluation of the RCM mechanism performance

Three groups of experiments were performed to test *e*_*i*_. Groups 1 and 2 were used to test *e*_*i*_ when the rotating and symmetrical-rod joints moved separately, and group 3 was used to test *e*_*i*_ when the laparoscope moved in a defined trajectory achieved by coordinated motion of the rotating and symmetrical-rod joints. Figure 9 shows the experimental setup. We measured the distances *l*_*pi*_, *l*_*ci*_, *k*_*pi*_, and *k*_*ci*_ and used two cell phones to take photographs in every step. We read the projections *R*_*ix*_ and *R*_*iy*_ from the coordinate sheets in the photographs.

**Fig. 10:**
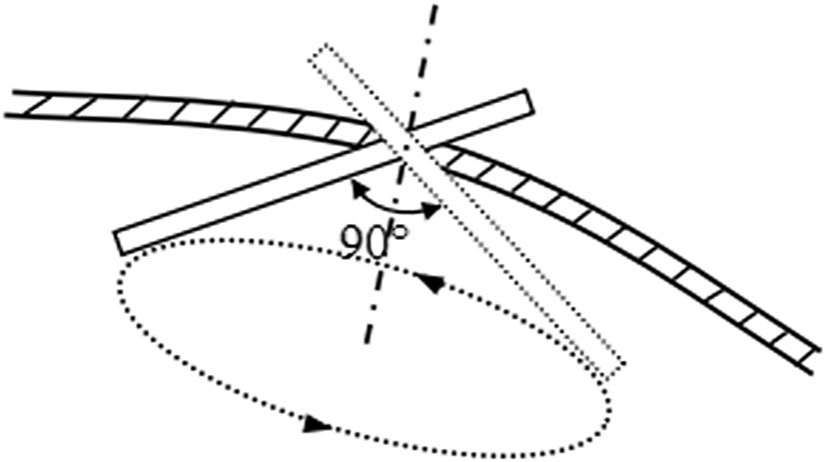
Illustration of tube movement in group 3 experiment

The movement ranges of the rotating and symmetrical-rod joints in the group 1 and 2 experiments were both − 70° to + 70°, and the position of the testing point was collected for every 5° rotation of the joints. Figure 10 illustrates the results of *e*_*i*_ between the collected and initial positions.

According to surgeons, in surgery, the laparoscope usually rotates in a range where the angle formed by the laparoscope axis and the normal of the incision port is less than 45°. Thus, in the group 3 experiment, we defined the tube movement such that the tube moved around the incision port, and the spatial angle between the tube and the normal of the incision port was 45°. Accordingly, the traces of the tube formed a cone, and the trajectory of the tube tip was a circle (Fig. 10). This tube movement was achieved through coordinated motion of the rotating and symmetrical-rod joints. Hence, according to the trajectory of the tube tip, the ranges of the joint movement were calculated using the above mentioned inverse kinematics solution and the joint movements were divided into 52 steps.

**Fig. 11:**
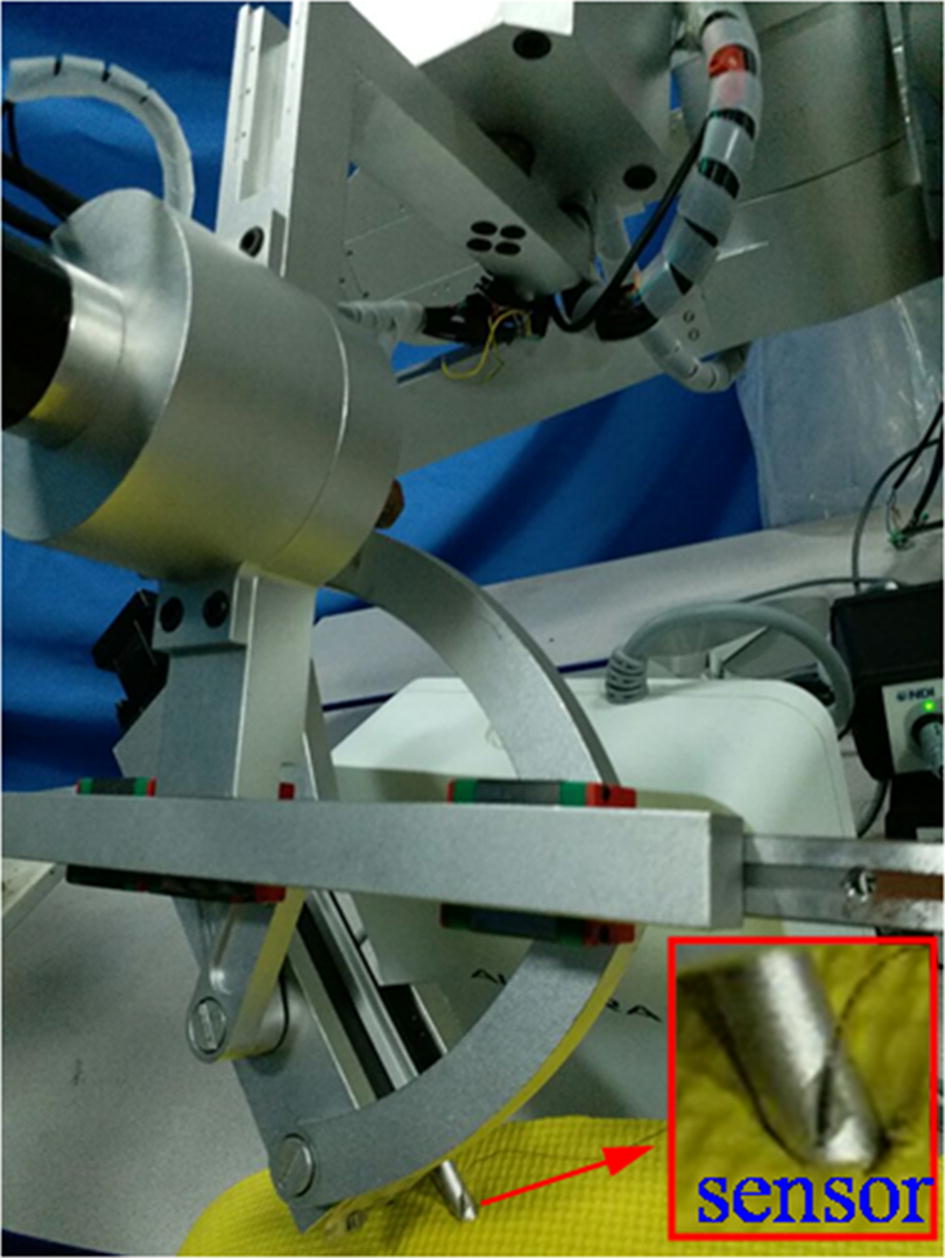
The experimental picture of the imitative experiment

As mentioned above, during surgery, to alleviate patient skin stress, the fixed point achieved by the mechanical structure of RCM mechanism is set to keep the coincidence with the incision port, to evaluate the mechanism “certring” performance under the action of incision port, an imitative experiment was carried out by measuring the position deviation of the fixed point. As shown in Fig. 11, an elastic cushion was adopted to represent the skin, the tube stood for laparoscope was inserted through the cushion, NDI Auraro testing system was employed and its sensor was stuck on the cylindrical surface of the fixed point, thus the position of the point stuck on sensor can be captured by the electromagnetic tracking system even under obstructions. Due to the restriction of the Auraro magnetic field, the position deviations were tested when the rotating and symmetrical-rod joints moved separately. The sensor collecting positions were the positions of the point on the cylindrical surface centred on fixed point, to observe the deviations of the fixed point position, they were converted to the positions of the fixed point.

## Results

**Fig. 12:**
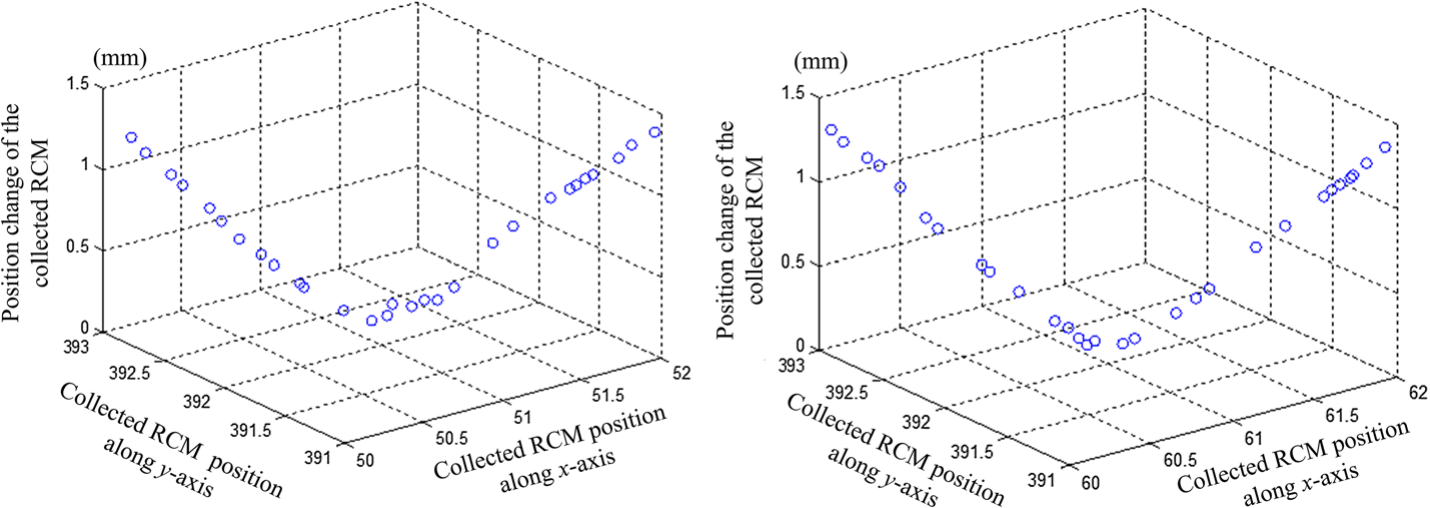
Position deviations in group 1 and 2 experiments

**Fig. 13:**
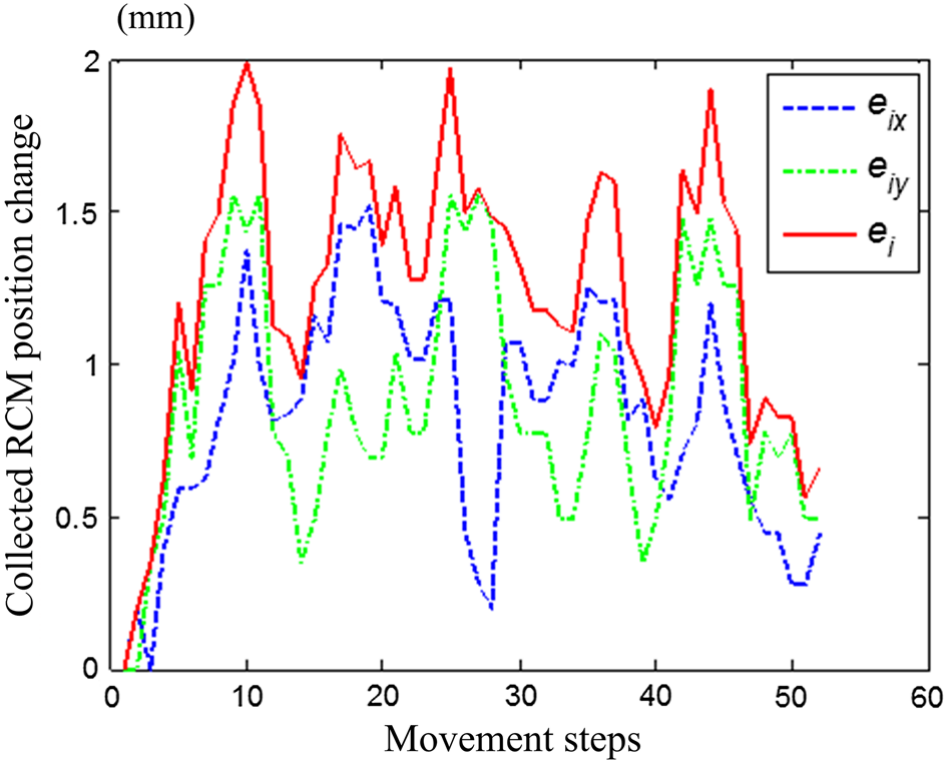
Position deviations in group 3 experiment

The experimental results in Fig. 12 showed that the position deviations of the testing point in the group 1 and 2 experiments were not greater than 1.5 mm while the joints moved from the zero position to the left and right limited positions, and Fig. 13 showed the position deviation and its components in group 3 in every step.

**Fig. 14:**
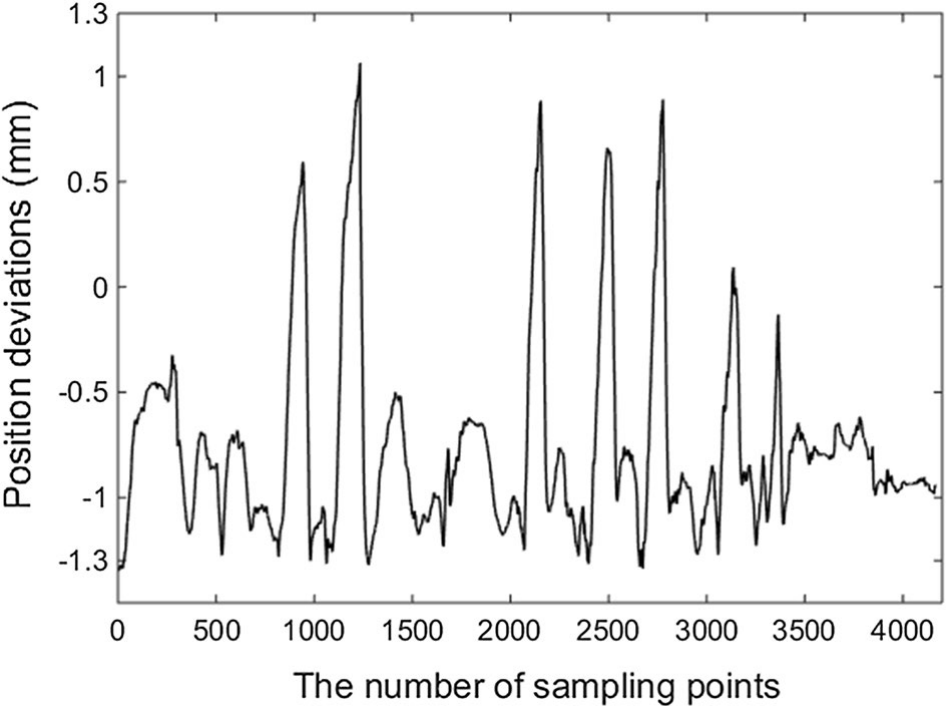
Position deviations in the imitative experiment

With the NDI Auraro testing system, after the calculation of the position deviations, the results were shown in Fig. 14. The position deviations of the fixed point were no more than 1.3 mm, which was smaller than that measured by cameras images. That because the elastic cushion provided a constraint for the movement backlash of the mechanism, which avoided the error caused by the joints backlash; in addition, the distance measurement and image processing involved in the cameras testing method may introduce errors. The test results of the two test methods are relatively close, which can reflect the performance of the proposed mechanism in achieving RCM-based motions.

According to results of the experiments validation, the position deviations fluctuated for approximately 1 mm, but did not exceed 2 mm. The proposed RCM mechanism was used herein to hold the laparoscop for providing surgeon visual display, for the wild field of view in RMIS, the 2 mm error of laparoscope position won’t affect surgeon observation. Meanwhile, the pressure on the incision port caused by the laparoscope position deviation was acceptable considering that the body surface tissue was sufficiently elastic to bear a 2-mm deviation, thus the proposed RCM mechanism met the RMIS requirement. In addition, the proposed RCM mechanism was a proof-of-principle prototype. The hinge points suffered from backlash because of the machining and assembly errors, and the motion error was mainly caused by the lack of machining accuracy. These problems could be overcome by improving the structural accuracy of the mechanism.

## Conclusion

In this paper, a new RCM mechanism for holding a laparoscope in RMIS was proposed, which consists of a symmetrical-rod joint, an axis-driving joint, and a linear joint for the laparoscope to achieve two RCM-based motions and insertion, respectively. The symmetrical-rod joint was designed using the characteristics of the symmetrical structure; thus, the RCM point is symmetrical to a fixed point so as to achieve ‘fixed’ RCM performance to alleviate the pressure caused by the surgical instrument. Thus, the laparoscope can pitch and yaw around the incision port. The proposed mechanism has high rigidity, as well as having a small volume due to the planar assembly mode. The entire mechanism is primarily composed of straight rods, which are easily machined. Moreover, because of its compact structure, the proposed mechanism can be applied to multi-robotic arms, effectively preventing collisions between the arms.

To simplify the kinematics calculation, the inverse kinematics was calculated from the perspective of the structural function, but not the mechanical structure; this greatly reduced the number of calculations and considerably aided control. As the function of the RCM mechanism is to provide instrument rotation around the RCM point, the RCM position deviation was selected as an index to verify the performance of the proposed mechanism. The deviations were tested by examining the position projection with two cameras and by using NDI Auraro testing system, respectively. The testing results show that the position deviations did not exceed 2 mm, which is lower than the 7 mm “certring” error of the RCM mechanism used in da Vinci surgical robot system [52]. The proposed RCM mechanism can achieve the RCM-based motion and meet the RMIS requirements, and its compact and simple structure can help to prevent collision between the robotic arms.
